# Supplementary material for: Nevirapine Biotransformation Insights: An Integrated In Vitro Approach Unveils the Biocompetence and Glutathiolomic Profile of a Human Hepatocyte-Like Cell 3D Model
Source: Int J Mol Sci. 2020 Jun 3;21(11):3998. doi: 10.3390/ijms21113998 (PMC7312429; doi:10.3390/ijms21113998)
Supplement: Supplementary file 1 [file ijms-21-03998-s001.pdf]

SUPPLEMENTARY MATERIALS

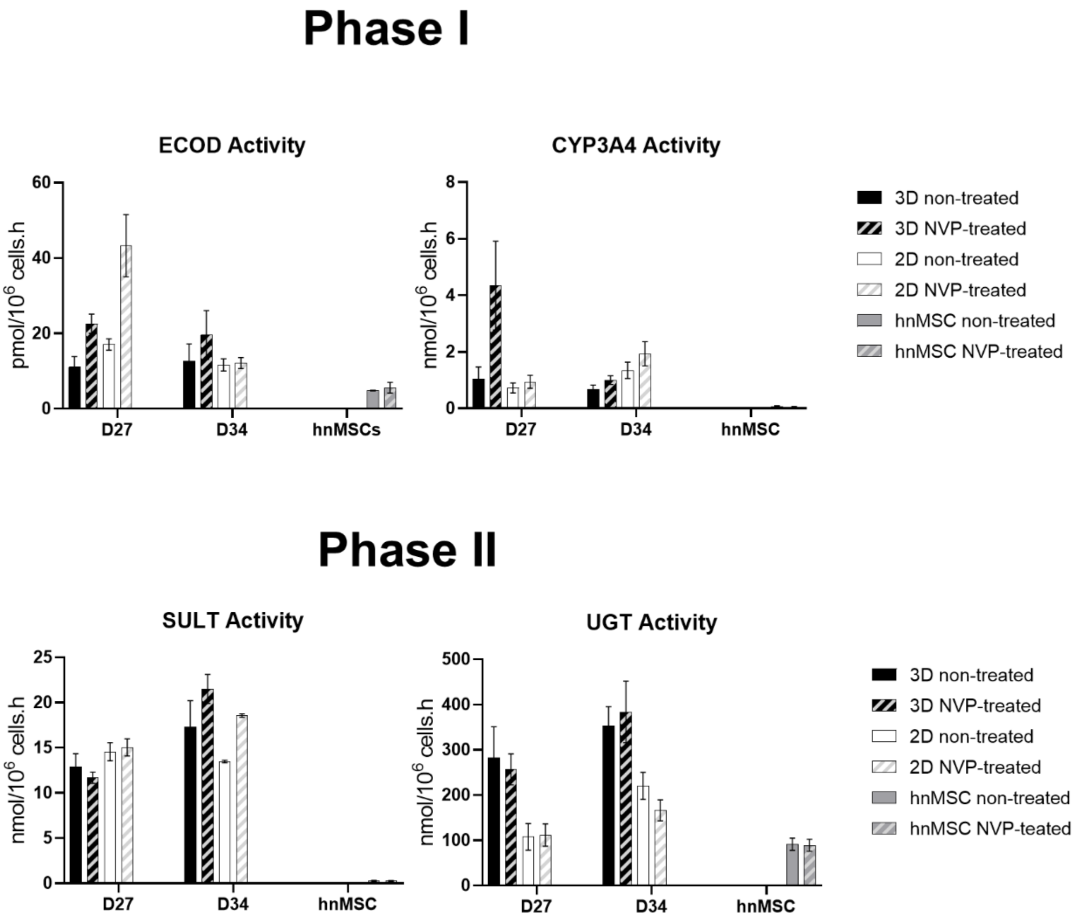

Supplementary Figure 1. Phase I and phase II enzymatic induction in 3D- and 2D-HLCs non-treated and after 3 (D27) and 10 days (D34) of NVP treatment. The data for hnMSC, cultured in monolayer and exposed to NVP for 3 days, are presented as negative control. All results are presented as the mean ± SD of at least three independent experiments.

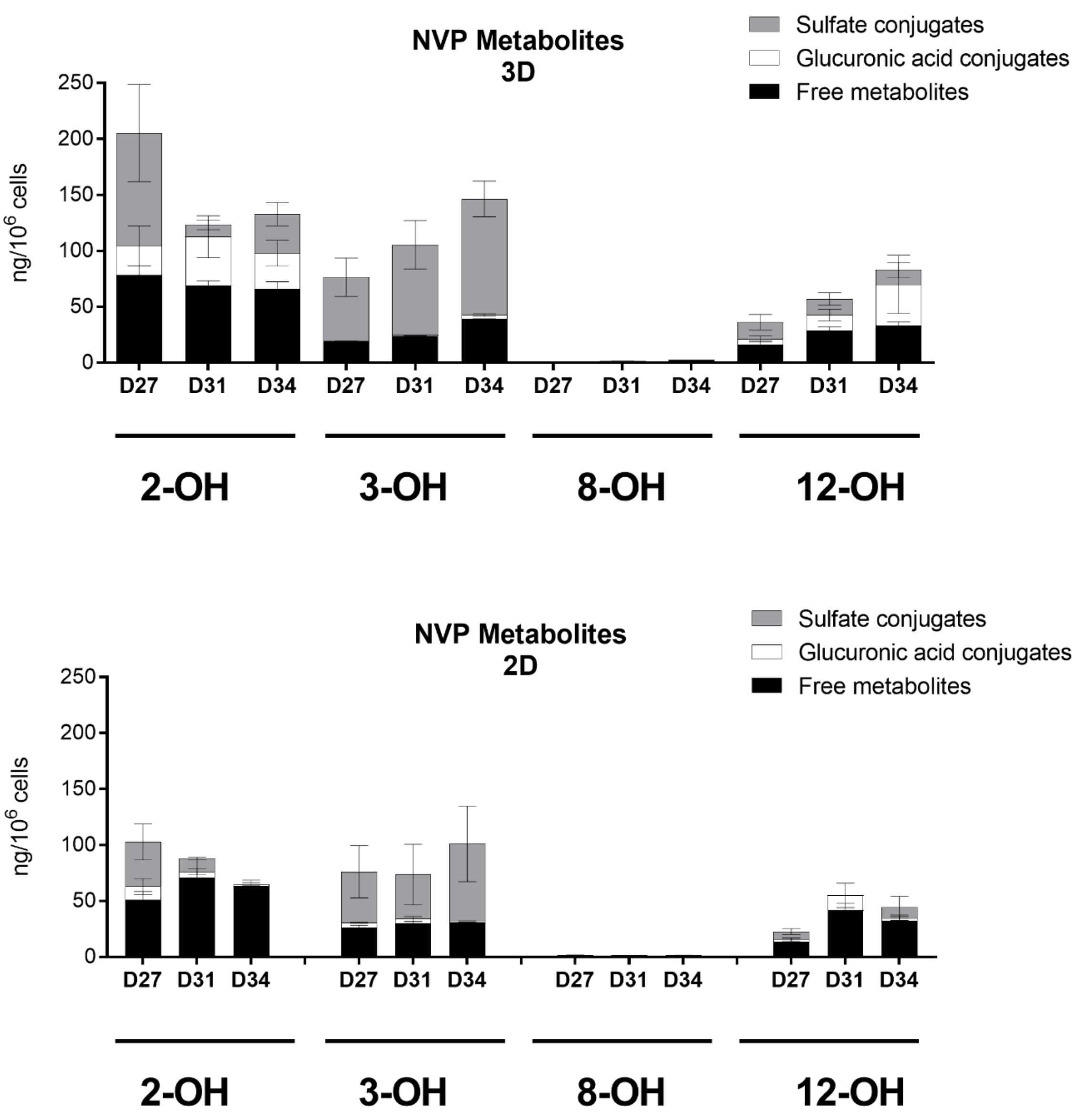

Supplementary Figure 2. Levels of NVP Phase I and II metabolites in 3D-HLCs (top) and 2D-HLCs (bottom) after 3 (D27), 7 (D31) and 10 days (D34) of NVP treatment. NVP metabolites are presented as free metabolites + sulfate conjugates + glucuronic acid conjugates. The data (Mean ± SD, n = 3) are normalized to total cell number.
